# DNA Methylation Causes Predominant Maternal Controls of Plant Embryo Growth

**DOI:** 10.1371/journal.pone.0002298

**Published:** 2008-05-28

**Authors:** Jonathan FitzGerald, Ming Luo, Abed Chaudhury, Frédéric Berger

**Affiliations:** 1 Chromatin and reproduction Group, Temasek Life Sciences Laboratory, National University of Singapore, Department of Biological Sciences, Singapore, Singapore; 2 Commonwealth Scientific and Industrial Research Organization, Black Mountain Laboratories, Black Mountain, Australian Capital Territory, Australia; University of California, Davis, United States of America

## Abstract

The parental conflict hypothesis predicts that the mother inhibits embryo growth counteracting growth enhancement by the father. In plants the DNA methyltransferase MET1 is a central regulator of parentally imprinted genes that affect seed growth. However the relation between the role of MET1 in imprinting and its control of seed size has remained unclear. Here we combine cytological, genetic and statistical analyses to study the effect of MET1 on seed growth. We show that the loss of MET1 during male gametogenesis causes a reduction of seed size, presumably linked to silencing of the paternal allele of growth enhancers in the endosperm, which nurtures the embryo. However, we find no evidence for a similar role of MET1 during female gametogenesis. Rather, the reduction of MET1 dosage in the maternal somatic tissues causes seed size increase. MET1 inhibits seed growth by restricting cell division and elongation in the maternal integuments that surround the seed. Our data demonstrate new controls of seed growth linked to the mode of reproduction typical of flowering plants. We conclude that the regulation of embryo growth by MET1 results from a combination of predominant maternal controls, and that DNA methylation maintained by MET1 does not orchestrate a parental conflict.

## Introduction

In flowering plants, meiosis is followed by the production of haploid structures, the male pollen and the female embryo sac, each containing two gametes. After double-fertilization, the female gametes, the egg cell and central cell, respectively give rise to the embryo and its nurturing annex, the endosperm. The embryo and the endosperm develop within the maternally derived seed integuments. Seed size is controlled primarily by interactions between the endosperm and integuments [1], [2] although the embryo also contributes [3].

The parental contributions to seed size were identified in crosses involving diploid and tetraploid plants. Tetraploid mothers produced smaller seeds when crossed to diploid fathers, however tetraploid fathers crossed to diploid mothers produced larger seeds [4], [5]. Hence seed size is enhanced by an excess of paternal genomes and restricted by an excess of maternal genomes. These phenomena were linked to the DNA methyltransferase *MET1*, using a dominant antisense construct, *MET1a/s* 6–9. Maternal inheritance of *MET1a/s* causes an increase of seed size whereas paternal inheritance has an opposite effect. MET1 is a key player in the control of parental genomic imprinting, which restricts gene expression from one of the two parental alleles [10]. In *Arabidopsis*, it was proposed that MET1 controls the expression of two pools of imprinted genes: maternally expressed inhibitors and paternally expressed enhancers of endosperm growth [11]. In *Arabidopsis* two imprinted genes dependent on MET1 have been identified [12]. MET1 silences the genes *FWA* and *FERTILIZATION INDEPENDENT SEED 2* (*FIS2*) in the male gametes [12]. *FIS2* and *FWA* are expressed in the female central cell [9], [13]. After fertilization *FIS2* and *FWA* are expressed in the endosperm from their maternal allele, while *MET1* maintains silencing on the paternal allele [12], [13]. The parental imbalance of expression thus defines *FIS2* and *FWA* as imprinted genes.

It was expected that the contrasting effects of *MET1a/s* were mediated by removal of silencing of the paternal allele of endosperm growth inhibitors, thus causing seed size increase and vice versa [11]. However, *MET1a/s* has a dominant effect, which does not allow distinguishing whether seed size variations in wild type (wt)×*MET1a/s* crosses originated from the loss of MET1 in the previous parental generation (sporophyte) or in the haploid generation producing the gametes (gametophyte). In addition, *MET1a/s* lines accumulate epimutations [6] and abnormal methylation profiles [14], which could be partially responsible of the phenotypes observed. A study based on a recessive loss-of-function allele, *met1-6* [15] showed clearly that the loss of *met1* during male gametogenesis reduces seed size. This result was also in agreement with the demonstration of a gametophytic effect of *met1-3* on the silencing of the paternal alleles of the imprinted genes *FIS2* and *FWA* [12]. However the existence of a gametophytic maternal effect of *met1-6* on seed size remained unclear [15] and a potential effect on *met1-6* loss of function on the diploid parental sporophytic generation was not tested explicitly. To address these concerns, we restricted our analysis to homozygous and heterozygous mutants derived from a self-fertilized heterozygous *met1-3/+* mother and compared the effects on seed development of *met1-3* loss of function during male gametogenesis, female gametogenesis and the parental diploid generation.

## Results and Discussion

### A distinctive paternal effect is associated to MET1 loss-of-function during male gametogenesis

**Figure 1:**
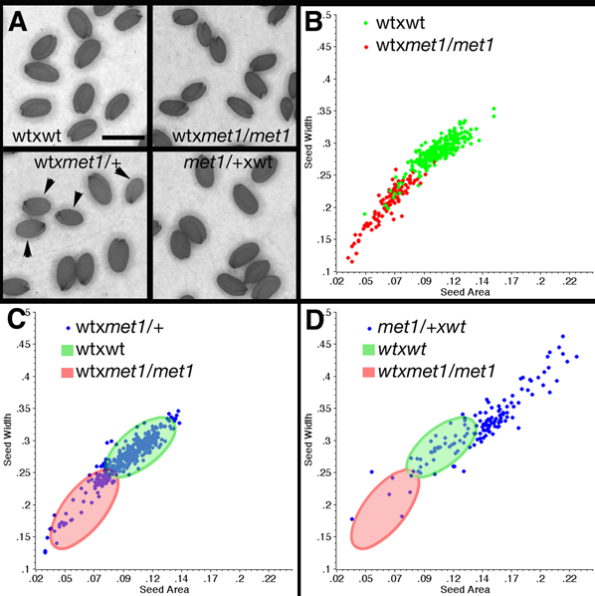
Parental effect of *met1-3*/+ on seed size. (A) Seed populations produced by crosses between wild-type (wt) ovules and pollen from *met1-3*/+ or *met1-3/met1-3* plants. The scale bar represents 0.5 mm. (B) Morphometric parameters of seeds from crosses between wt ovules and pollen from wt or from *met1/met1* plants. (C) Morphometric parameters of seeds from crosses between ovules from wt plants and *met1-3*/+ pollen. The green and red ovals represent the extent of the populations of seeds shown in B. (D) Morphometric parameters of seeds from crosses between ovules from *met1*/+ plants and wild-type pollen. The green and red ovals represent the extent of the populations of seeds shown in B.

The null recessive allele *met1-3* causes a loss of DNA methylation in first generation homozygous plants [16]. The loss of *met1* function is caused by a T-DNA insert linked to a gene conferring resistance to the herbicide BASTA. To confirm specific parental contributions of *met1-3* to seed size, we analyzed digital images of seeds from crosses that varied MET1 genotype and parent of transmission (Figure 1, Table 1). Seeds produced by crosses between wild-type ovules and pollen from *met1-3/met1-3* plants were smaller than seeds produced between wild type ovules and wild type pollen (Figure 1A). Quantitative analysis resolved these two genotypes into two distinct populations based on seed width and length (n = 108; P<.0001 for ANOVA, t-test and Mann Whitney) (Figure 1B, Table 1). This verified that *met1-3* has a paternal effect on seed size as observed in previous studies [7], [9], [15]. We then conducted the same experiment with heterozygous *met1-3/+* plants. Half of the pollen from *met1-3/*+ plants carries the *met1-3* allele causing re-activation of imprinted genes [12] and other silenced loci [17]. It is thus possible to predict a gametophytic paternal effect of *met1* with size reduction in only 50% of the seeds produced by wild type ovules crossed to *met1-3/*+ pollen. Accordingly, we observed both large and small seeds by visual inspection (Figure 1A; 45.4% small seeds; n = 900) and quantitative analysis (wt×*met1/*+, n = 374; wt×wt, n = 257; P<.0001 for ANOVA, t-test and Mann Whitney) (Figure 1C, Table 1). In the small seeds from crosses between wild type ovules and pollen from *met1-3/+* plants, embryo growth was relatively normal as compared to the endosperm, which exhibited reduced growth (Figures S1 A and S1B, see the supplemental data available with this article online).

**Table 1:** Morphometric measurements of seeds from various crosses reported in Figure 1

Cross genotype (mat×pat)	n	Seed area mean	s.d.	s.e.m	Seed width mean	s.d.	s.e.m
wt×wt	257	0.105	0.018	0.001	0.283	0.033	0.002
wt×*met1/met1*	108	0.65	0.014	0.001	0.207	0.031	0.003
wt×*met1*/+	374	0.98	0.022	0.001	0.273	0.036	0.002
*Met1*/+×wt	138	0.142	0.037	0.003	0.321	0.056	0.005

**Figure 2:**
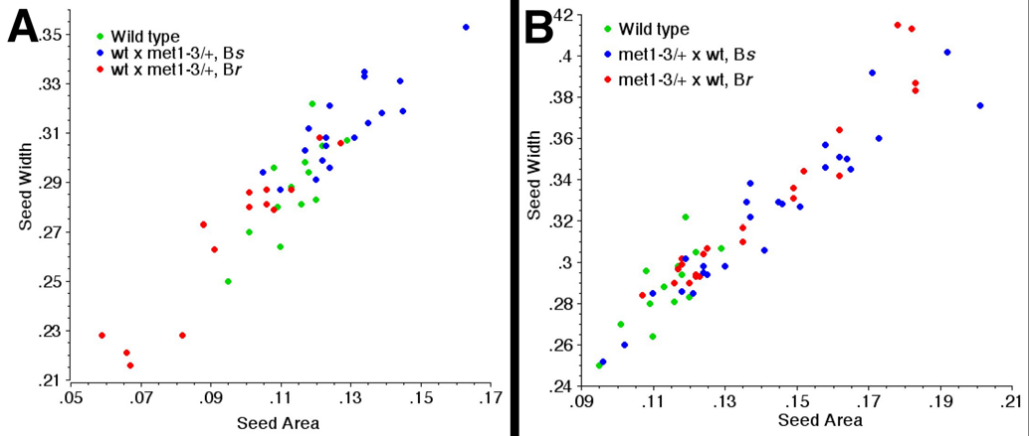
Correlation between seed size and the inheritance of *met1-3* associated to BASTA resistance. (A) BASTA resistance (B*r*) and sensitivity (B*s*) are correlated with seed size in seeds from crosses between wild-type ovules and *met1-3*/+ pollen. Segregation of the BASTA marker remains 1∶1 (p = 0.4795 χ^2^), so although some seed lethality was observed (n = 11) it is not linked to *met1-3*. (B) B*r* and B*s* are not correlated with seed size in seeds from crosses between *met1-3*/+ ovules and wild-type pollen.

To confirm the link between the small seeds and paternal inheritance of *met1-3*, seeds from wt×*met1-3*/+ crosses were visually sorted according to their size relative to a wild type control, and BASTA resistance associated to *met1-3* was tested. Two populations of seeds were distinguished. All smallest seeds were resistant to BASTA (n = 323) while all largest seeds were sensitive to BASTA (n = 336). The 1∶1 proportion supported the predicted association of the paternal effect of *met1-3* to gametogenesis (p = 0.6126 χ^2^). As we did not analyze the entire population we may have missed a complex genetic component regulating seed size. To ensure that abnormally small seeds or seed lethality were not missing from our bulked seed population, we analyzed all seeds from single crosses between wild-type mothers and pollen from *met1-3*/+ plants (Figure 2A, Table 2). In this analysis we also ensured that crosses with pollen from wt and *met1-3*/+ plants were performed on the same mother plant to allow an absolute size comparison. BASTA resistance correlated with the smallest seeds of the population (p<0.0001 ANOVA and Mann-Whitney) demonstrating that paternal inheritance of *met1-3* causes seed size reduction as a result of the loss of MET1 activity during male gametogenesis. The loss of MET1 during male gametogenesis may allow paternal expression of imprinted growth inhibitors and cause a decrease of endosperm and seed size. Loss-of-function paternal effects are uncommon and until now have only been linked to defects in fertilization in *Drosophila* [18], [19], *C.elegans* [20] and *Arabidopsis* [3]. We thus conclude that *met1-3* causes a paternal effect associated with defects after fertilization and thus representing a distinct class of paternal effect mutations.

**Table 2:** Morphometric measurements of seeds correlated to BASTA R as reported in

Cross Genotype		Seed Genotype								
		BASTA R			BASTA S					
		n	mean	s.d.	n	mean	s.d.	ANOVA	M-W	
mat wt×pat *met1-3*/+										
	area	14	0.095	0.021	18	0.128	0.014	<0.0001		<0.0001
	width	14	0.267	0.031	18	0.313	0.017	<0.0001		<0.0001
mat *met1-3*/+×pat wt										
Silique 1	area	13	0.122	0.007	12	0.121	0.013	0.7953		0.8066
	width	13	0.298	0.009	12	0.290	0.019	0.1787		0.3270
Silique 2	area	9	0.167	0.015	14	0.161	0.019	0.4847		0.4120
	width	9	0.368	0.033	14	0.352	0.024	0.1815		0.2567

Figure 2 and corresponding to the supplementary Figure S2.

### Loss of MET1 during female gametogenesis does not impact on seed size

While crosses between wild type ovules and the *MET1a/s* pollen caused a decrease of seed size, a symmetrical increase of seed size was observed in seeds from the reciprocal crosses *MET1a/s*×wt. [7]–[9]. We tested whether maternal inheritance of *met1-3* from *met1-3*/+ mothers would increase size in 50% of the seeds. Crosses between ovules from *met1-3*/+ plants and wild-type pollen did exhibit increased seed size relative to wild type controls (Figure 1A; n = 900) correlated with an increased in endosperm size (Figure S1 C, see the supplemental data available with this article online). However, this increase in size affected the whole population of seeds (Figure 1D, Table 1, n = 138). Largest seeds selected by visual inspection from a population of 900 seeds from wt×*met1-3*/+ crosses did not show a preferential resistance to BASTA (55.1% BASTA Resistant in a population of n = 84 largest seeds). This is contrary to the expected consequence of a maternal gametophytic effect of *met1-3*/+, which should produce a greater proportion BASTA resistance among the largest seeds in a population derived from *met1-3*/+×wt crosses. To confirm this finding we compared BASTA resistance and seed size in an entire population of seeds from *met1-3*/+×wt crosses from a single plant. We observed that larger seeds did not always inherit the *met1-3* allele and the means of size measurements did not differ between seed genotypes (Figure 2B, Table 2 and Figure S2, see the supplemental data available with this article online). These results were in clear contrast to the results obtained from crosses involving pollen from *met1-3*/+ plants. The inheritance of *met1-3* from *met1-3*/+ plants through the female gametes did not cause the increase of size in 50% of the seed population as expected for a gametophytic maternal effect. However we observed an overall increase of seed size in the entire population of seeds (Fig. 1D, Table 1). Thus, it was possible that either the gametophytic effect was not fully penetrant and could not be detected clearly. Alternatively it was possible that the maternal effect of *met1* was mediated from the maternal tissues surrounding the seed.

### Maternal effects linked to loss-of-function of MET1 in vegetative tissues

In seeds derived from *met1-3*/+ fathers we expected that genetically wild-type seeds would have a wild-type seed size. In contrast, both the seed area and width of the BASTA sensitive wild type seeds derived from *met1-3*/+ fathers are significantly larger than the wild-type controls pollinated after emasculation and grown in the same conditions, even though these seeds are genetically identical (Table 2). This effect on seed size likely originates from the reduced dosage of active MET1 in the heterozygous *met1-3*/+ vegetative tissues. Similarly the average seed size of wild type seed produced from crosses between ovules from *met1*/+ plants and wild type pollen were also larger than wild type ovules from controls emasculated wild type plants crossed with wild type pollen (Table 2). Since we failed to detect a gametophytic component in the genetic maternal control of seed size by *met1-3*/+ plants, we concluded that the size increase observed in *met1*/+×wt crosses originated from the effect of *met1* in vegetative tissues. Thus, plants heterozygous for *met1-3* enhanced seed growth both maternally and paternally with no evidence for antagonism between the two parents. In addition our results suggest that an overall reduction of MET1 levels in *met1-3*/+ plants could lead to a reduced level of DNA methylation activity prior to meiosis and promote seed size increase.

### MET1 controls embryo size through its action on the maternal tissues

**Figure 3:**
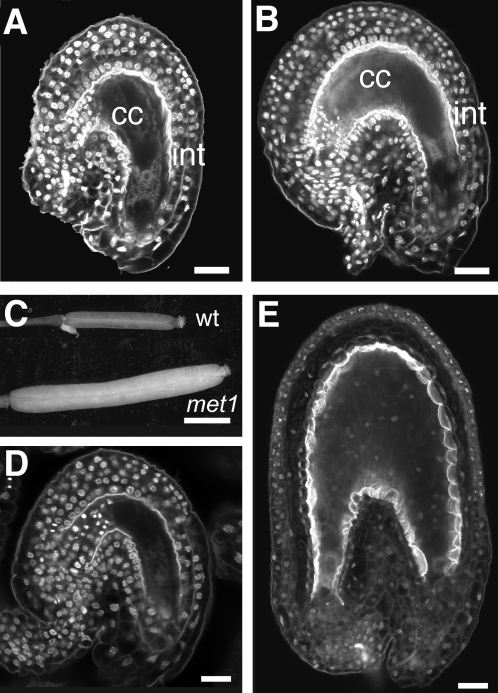
Maternal effects of *met1/met1* on ovule integument. (A) Wild-type ovule at the mature stage shows four or five cell layers of integuments (int) surrounding the central cell (cc). (B) A similar confocal section of a *met1/met1* ovule. (C) Fruits from *met1-3/met1-3* plants elongate in absence of fertilization (10 Days After Emasculation, (DAE)) in comparison to wild-type fruits. (D) Wild-type ovule with collapsed central cell at 8 DAE. (E) Seed-like structure in elongated fruits from *met1-3/met1-3* plants at 8 DAE. Scale bars represent 20 µm (A, B, D and E) and 1.5 mm (C).

The maternal inheritance of the dominant *MET1a/s* construct caused a dramatic increase of seed size [7]. Similarly, seeds from crosses between ovules from *met1-6* [15] or *met1-3* homozygous crossed to wild type pollen are much larger than seeds produced from *met1*/+ heterozygous mothers crossed to wild type pollen (Figures S1, C and D, see the supplemental data available with this article online). The range of phenotypes suggested that seed size and development were influenced by *MET1* dosage in the maternal sporophyte. All seeds were affected, indicating that defects could originate from the maternal tissues responsible for supplying maternal nutrients to the seed or the maternal seed integuments. Deregulation of cell proliferation and cell elongation of integuments influences seed size [1], [21], [22]. We thus investigated whether MET1 controls integuments development. We observed that *met1-3/met1-3* integuments contain 50% more cells than in the wild type (Figures 3A and 3B and Table 3). We thus conclude that MET1 represses cell proliferation in the integuments. In addition, we observed that in the absence of fertilization, the fruits of *met1-3/met1-3* plants elongated (Figure 3C and Table 3), resulting in production of seed-like structures devoid of embryo and endosperm (Figure 3, D and E and Table 3). Similar observations were made with *MET1a/s* plants (Table 3 and Figure S3, see the supplemental data available with this article online). The autonomous seed-like structures are devoid of endosperm or embryo and develop only from ovules that are deficient of MET1 in the sporophytic integuments but not from ovules from *met1*/+ plants, 50% of which are deficient of MET1 in the female gametophyte. We conclude that autonomous growth of seed-like structures did not originate from the loss of MET1 activity in the central cell or the egg cell. Rather, *MET1* thus controls seed size maternally through its action on cell proliferation and elongation in the seed integuments. Double fertilization causes enhanced cell division followed by elongation in the wild type [1]. Our results thus suggest that double-fertilization releases MET1-inhibited controls. Hence we show that mechanisms acting in the integuments in addition to the endosperm [23] and the embryo [3], [24] prevent seed development in absence of fertilization.

**Table 3:** Morphometric measurements of autonomous fruits and seeds produced by plants deficient for MET1.

Genotype	Fruit elongation at 10 DAP/11DAE			Autonomous seed-like structures at 8DAE			Integument cell number in the endothelium at 5 DAE			Integument length at 5 DAE		
	Length (mm)	s.d.	n	%	s.d.	n	#	s.d.	n	Length (µm)	s.d.	n
+/+	3.8	0.2	5	0	0	124	27.6	2.1	8	190.2	23.1	4
*met1-*3*/met1-3*	8.5	0.7	8	17.8	3.2	134	43.4	2.7	7	370.5	51.2	4
*MET1a/s*	8.9	0.3	20	13.1	5.5	355	41.6	2.5	8	366.2	49.5	4

### Conclusions

MET1 independently controls both endosperm growth and cell division and elongation of the integuments. Presumably MET1 silences maternal genes in the integuments and restricts seed growth through this maternal sporophytic control. In addition MET1 restricts the expression of imprinted genes in endosperm to the maternal alleles, resulting eventually in a different type of maternal control of endosperm growth. Our results also suggest that a memory of the maternal epigenetic status prior to meiosis is recorded during gametogenesis and influences seed size. Overall the epigenetic control of seed size by MET1 appears to result primarily from maternal controls. These derive directly from the action of MET1 on the sporophytic vegetative tissues and indirectly from the restriction of expression of imprinted inhibitors of seed size to their maternal allele by MET1 acting during male gametogenesis. This conclusion does not support MET1-mediated antagonism between imprinted loci expressed from the paternal or maternal genomes as originally predicted by the parental conflict hypothesis. It is unlikely that CMT3 and DRM2 involved in global de novo DNA methylation control seed size since they do not appear to impact the expression of imprinted genes [25], [26]. However we do not exclude that other epigenetic controls such as histone methylation by Polycomb group complexes [26]–[28] are responsible for an opposite action of the expression between paternally and maternally expressed imprinted genes. In mammals, the function and regulation of some imprinted genes support the parental conflict theory [11], [12], [17], [29]. However some results also suggest a predominant maternal control of placental and embryo growth [30]–[32]. In conclusion, in plants and mammals a complex series of maternal controls balance the unequal parental contributions to the offspring and may mimic a parental conflict without involving symmetrical antagonistic molecular controls.

## Materials and Methods

### Plant lines and growth conditions

The wild-type control lines C24 and Col were supplied by the ABRC stock center. The line *met1a/s* (C24) was supplied by J. Finnegan [6]. The line *met1-3* (Col) was supplied by J. Paszkowsky and contains a TDNA insert conferring resistance to BASTA [17]. The *met1-3* line was maintained as heterozygous by repeated backcrosses to wild-type plants in order to avoid accumulation of epigenetic defects. Once allowed to self, the resulting segregating homozygous plants were used for emasculation for crosses to wild-type plants and for observation of autonomous development.

Plants were grown at 22 C and 60% hygrometry in short days (16 h night) for three weeks followed by long days (8 h night) in Conviron Growth chambers.

### Microscopy and measurements

Developing seeds were cleared with Hoyer's medium and observed with DIC optics with a Leica microscope (DM600). Images were recorded with a Snapshot camera and processed with Metamorph for morphometric measurements. For confocal microscopy, material was prepared and observed as described previously [1].

### Experimental strategy

In order to evaluate the relationship between seed size and parental inheritance of *met1* we performed a series of four experiments. We produced crosses between wild type and *met1-3*/+ plants grown in the same conditions and obtained two populations of 900 seeds with inheritance of *met1* from the mother or from the father. We visually separated seeds according to size categories in each population and tested BASTA resistance in a subset representing the largest or smallest seeds. In a second series of crosses we produced crosses between wild type emasculated plants and wild-type or *met1-3*/+ plants or *met1-3/met1-3* plants grown together. The seeds obtained were imaged and seed size was measured as detailed below and the data are reported in Figure 1 and Table 1. We obtained a third series of crosses from single plants in order to have an ideal wild type control to compare seed size with and to establish correlation with BASTA resistance. The dataset is reported in Figure 2, Figure S2 and Table 2.

### Statistical Analysis

To determine seed area and height, digital images of seeds on a white background were thresholded in Adobe Photoshop CS2 (an example of such outline is shown in Figure S2). These black and white images were analyzed by ImageJ. We set a threshold on the grayscale such that the seed appears uniformly black against a white background. The black areas are detected automatically and converted as ellipsoids with the measurement of area and minor axes. To test the differences between the means of two seed populations, both analysis of variation (ANOVA) and the non-parametric Mann-Whitney test (M-W) were employed as certain portions of very small seeds in some experiments may have violated the normality assumption in ANOVA. 1∶1 ratios of small and large seeds were tested by the Pearson's χ^2^ test (χ^2^). Finally, we used the Kalmagorov-Smirnoff Normality test (K-S) to determine whether seed size phenotypes fit a normal distribution based on comparison to a generated ideal normal distribution of similar mean and standard deviation. Calculations were performed using StatView 5.0.1 (SAS Institute, Cary NC), except for χ^2^, which was calculated on Excel×(Microsoft). p-values provided in the text are followed by the abbreviation of the test used.

## Supporting Information

Figure S1 Parental effect of met1-3/+ on endosperm size during seed development. Endosperm size was measured at 3 DAP (A, B) and at 6 DAP (C, D) in wild-type seeds (A, C), in seeds resulting from crosses between wild-type ovules and pollen from met1-3/+ plants (B, D). Scale bars represent 20 µm (A, B) and 50 µm (C, D). Cytological observations were performed to establish the origin of the reduction of seed size caused by paternal inheritance of met1. The final seed size depends both on the extent of cell proliferation in the embryo and on the degree of endosperm growth during the early phase of seed development 1 to 4 Days After Pollination (DAP). Until the late heart stage we did not observe any reduction of cell proliferation in the embryo of seeds, which inherit met1 paternally or maternally. Patterns of embryo development did not show obvious modifications and met1/+ embryos were viable. In contrast seeds which inherited met1 paternally showed a reduction of endosperm size as early as the beginning of the embryo globular stage. Early endosperm development is characterized by a series of nuclei divisions, not followed by cell divisions leading to a syncytium. The frequency and number of syncytial divisions were not altered by paternal inheritance of met1 and both smaller met1/+ seeds and larger wild-type seeds contained approximately 100 nuclei as expected at 3 DAP. The difference in size between the two populations of seeds increased during development leading to two easily distinguishable classes.
(0.49 MB DOC)

Figure S2 Parental effect of met1-3/+ ovules crossed to wild-type pollen on seed size during seed development, correlated with BASTA resistance (R) or sensitivity (S).
(1.73 MB PDF)

Figure S3 Autonomous development of fruit and seed in MET1a/s. (a) Increase of fruit elongation in MET1a/s plants in absence of fertilization 7 Days After Emasculation (DAE). (b) Autonomous development of ovules in met1a/s silique in absence of fertilization (7 DAE). (c) Cleared autonomous seed from MET1a/s plant 7 DAE show remnants of the central nucleus and the egg cell. (d) Wild-type seed (7 DAE). Bars represent 0.8 mm (a), 0.5 mm (b), and 40 µm (c, d).
(0.94 MB PDF)
