# Supplementary material for: DNA Methylation Causes Predominant Maternal Controls of Plant Embryo Growth
Source: PLoS One. 2008 May 28;3(5):e2298. doi: 10.1371/journal.pone.0002298 (PMC2390113; doi:10.1371/journal.pone.0002298)
Supplement: Figure S1 — Parental effect of met1-3/+ on endosperm size during seed development. Endosperm size was measured at 3 DAP (A, B) and at 6 DAP (C, D) in wild-type seeds (A, C), in seeds resulting from crosses between wild-type ovules and pollen from met1-3/+ plants (B, D). Scale bars represent 20 µm (A, B) and 50 µm (C, D). Cytological observations were performed to establish the origin of the reduction of seed size caused by paternal inheritance of met1. The final seed size depends both on the extent of cell proliferation in the embryo and on the degree of endosperm growth during the early phase of seed development 1 to 4 Days After Pollination (DAP). Until the late heart stage we did not observe any reduction of cell proliferation in the embryo of seeds, which inherit met1 paternally or maternally. Patterns of embryo development did not show obvious modifications and met1/+ embryos were viable. In contrast seeds which inherited met1 paternally showed a reduction of endosperm size as early as the beginning of the embryo globular stage. Early endosperm development is characterized by a series of nuclei divisions, not followed by cell divisions leading to a syncytium. The frequency and number of syncytial divisions were not altered by paternal inheritance of met1 and both smaller met1/+ seeds and larger wild-type seeds contained approximately 100 nuclei as expected at 3 DAP. The difference in size between the two populations of seeds increased during development leading to two easily distinguishable classes. (0.49 MB DOC) [file pone.0002298.s001.pdf]

met1-3/+ ♀ X wild type ♂

Silique 1

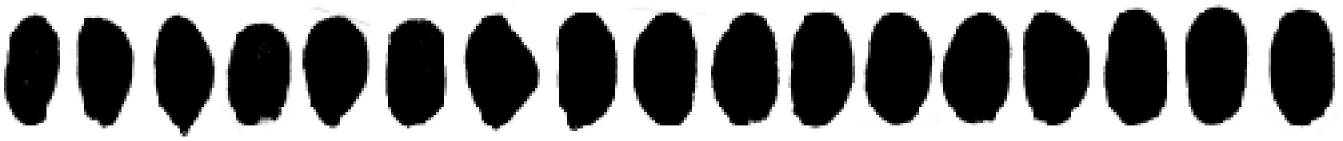

Area(mm2)	0.094	0.096	0.102	0.105	0.107	0.11	0.114	0.115	0.116	0.117	0.118	0.118	0.118	0.119	0.12	0.121	0.122
Width(mm)	0.242	0.252	0.26	0.285	0.284	0.285	0.303	0.279	0.29	0.297	0.299	0.302	0.286	0.302	0.29	0.285	0.294
Basta	X	S	S	X	R	S	X	X	R	R	R	R	S	S	R	S	R

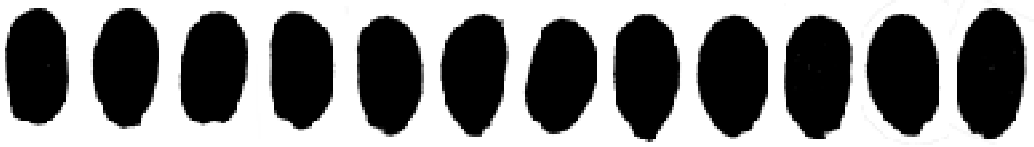

0.122	0.123	0.124	0.124	0.124	0.125	0.125	0.13	0.135	0.135	0.137	0.141
0.293	0.293	0.295	0.304	0.298	0.294	0.307	0.298	0.31	0.317	0.322	0.306
R	R	S	R	S	S	R	S	R	R	S	S

Silique 2

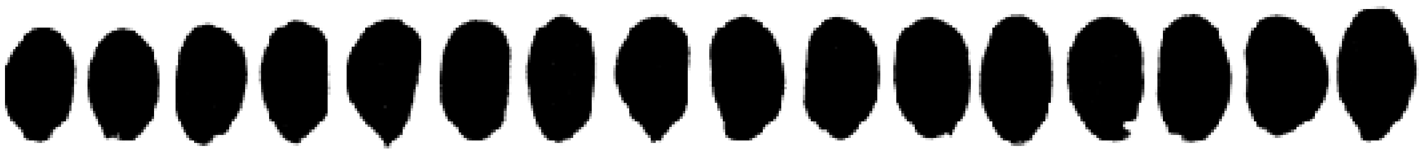

Area(mm2)	0.136	0.137	0.145	0.146	0.149	0.149	0.151	0.152	0.158	0.158	0.162	0.162	0.162	0.164	0.165	0.171	0.173
Width(mm)	0.329	0.338	0.329	0.328	0.336	0.331	0.327	0.344	0.357	0.346	0.351	0.364	0.342	0.35	0.345	0.392	0.36
Basta	S	S	S	S	R	R	S	R	S	S	S	R	R	S	S	S	S

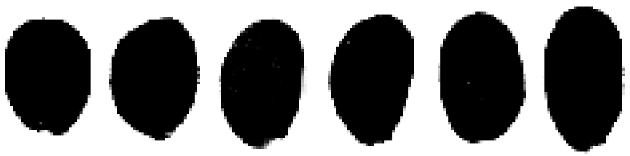

0.178	0.182	0.183	0.183	0.192	0.201
0.415	0.413	0.383	0.387	0.402	0.376
R	R	R	R	S	S

Figure S1: Parental effect of met1-3/+ ovules crossed to wild-type pollen on seed size during seed development, correlated with Basta resistance (R) or sensitivity (S)
