# Supplementary figures and images for: DNA Methylation Causes Predominant Maternal Controls of Plant Embryo Growth
Source: PLoS One. 2008 May 28;3(5):e2298. doi: 10.1371/journal.pone.0002298 (PMC2390113; doi:10.1371/journal.pone.0002298)

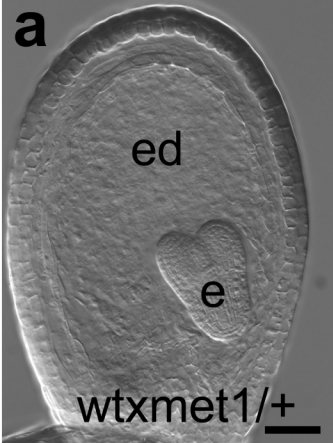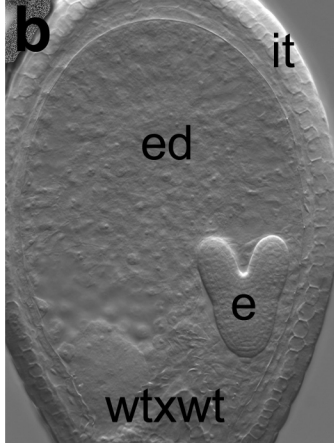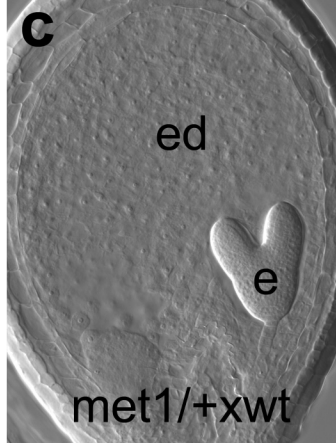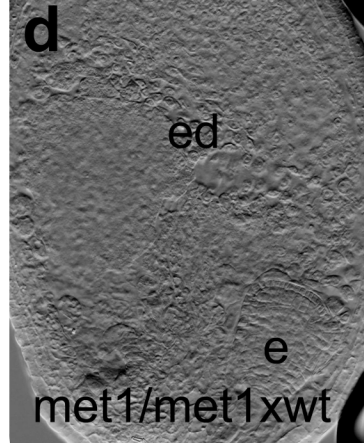

FitzGerald Supplementary Figure 2

Supplement: Figure S2 — Parental effect of met1-3/+ ovules crossed to wild-type pollen on seed size during seed development, correlated with BASTA resistance (R) or sensitivity (S). (1.73 MB PDF) [file pone.0002298.s002.pdf]

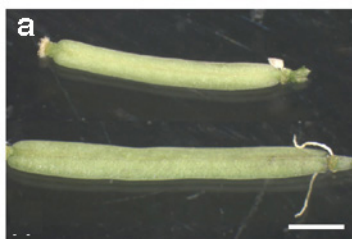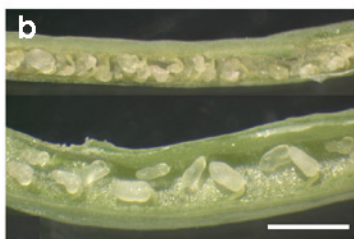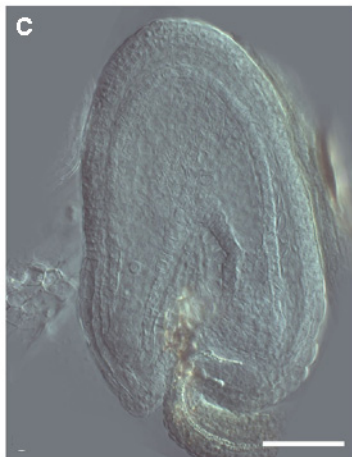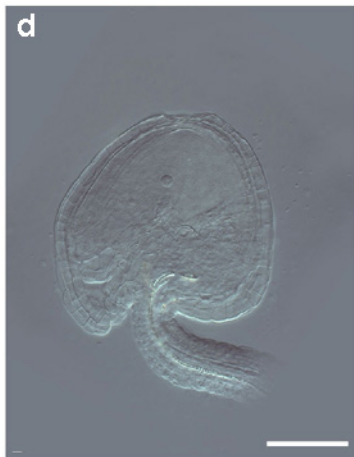

**FitzGerald Supplementary Fig. 3**

Supplement: Figure S3 — Autonomous development of fruit and seed in MET1a/s. (a) Increase of fruit elongation in MET1a/s plants in absence of fertilization 7 Days After Emasculation (DAE). (b) Autonomous development of ovules in met1a/s silique in absence of fertilization (7 DAE). (c) Cleared autonomous seed from MET1a/s plant 7 DAE show remnants of the central nucleus and the egg cell. (d) Wild-type seed (7 DAE). Bars represent 0.8 mm (a), 0.5 mm (b), and 40 µm (c, d). (0.94 MB PDF) [file pone.0002298.s003.pdf]
